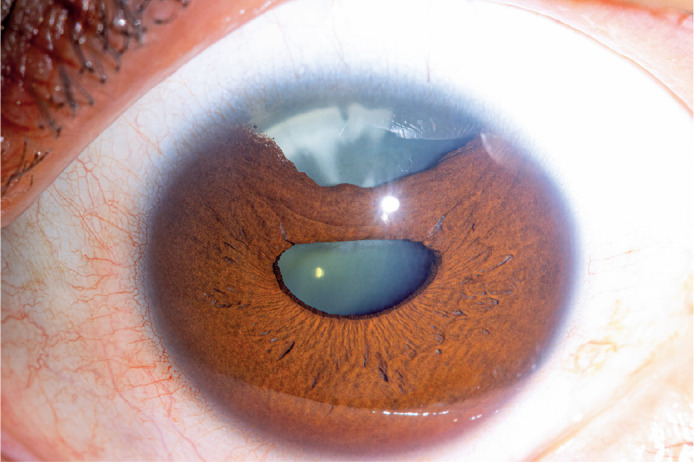# Iridodialysis

**DOI:** 10.5935/0004-2749.2025-0092

**Published:** 2025-06-24

**Authors:** Gilberto Krasilchik, Nicole Bulgarão Maricondi Almeida, Newton Kara-Junior

**Affiliations:** 1 Ophthalmology Department, Faculdade de Medicina, Santa Casa de Misericórdia de São Paulo, São Paulo, SP, Brazil; 2 Ophthalmology Department, Hospital das Clínicas, Universidade de São Paulo, São Paulo, SP, Brazil

Iridodialysis refers to the separation of the iris from its attachment to the ciliary body. This condition commonly arises due to trauma or complications during intraocular surgery, given the inherently weak structure of the iris root^(^1^)^. The iris plays a crucial role in controlling pupil diameter^(^1^)^. Consequently, iris defects can lead not only to cosmetic deformations, such as polycoria and ectopic pupil, but also to significant functional impairments. These include monocular diplopia, photophobia^(^2^)^, reduced visual quality, severe glare, and decreased contrast sensitivity^(^1^)^.

**Figure:**